# Evolutionary divergence of 3’ UTRs in cichlid fishes

**DOI:** 10.1186/s12864-018-4821-8

**Published:** 2018-06-05

**Authors:** Peiwen Xiong, C. Darrin Hulsey, Axel Meyer, Paolo Franchini

**Affiliations:** 1 Chair in Zoology and Evolutionary Biology, Department of Biology, University of Konstanz, 78457 Konstanz, Germany; 2 Radcliffe Institute for Advanced Study, Harvard University, Cambridge, MA 02138 USA

**Keywords:** Adaptive radiation - Comparative genomics - Regulatory evolution - miRNA binding sites

## Abstract

**Background:**

Post-transcriptional regulation is crucial for the control of eukaryotic gene expression and might contribute to adaptive divergence. The three prime untranslated regions (3’ UTRs), that are located downstream of protein-coding sequences, play important roles in post-transcriptional regulation. These regions contain functional elements that influence the fate of mRNAs and could be exceptionally important in groups such as rapidly evolving cichlid fishes.

**Results:**

To examine cichlid 3’ UTR evolution, we 1) identified gene features in nine teleost genomes and 2) performed comparative analyses to assess evolutionary variation in length, functional motifs, and evolutionary rates of 3’ UTRs. In all nine teleost genomes, we found a smaller proportion of repetitive elements in 3’ UTRs than in the whole genome. We found that the 3’ UTRs in cichlids tend to be longer than those in non-cichlids, and this was associated, on average, with one more miRNA target per gene in cichlids. Moreover, we provided evidence that 3’ UTRs on average have evolved faster in cichlids than in non-cichlids. Finally, analyses of gene function suggested that both the top 5% longest and 5% most rapidly evolving 3’ UTRs in cichlids tended to be involved in ribosome-associated pathways and translation.

**Conclusions:**

Our results reveal novel patterns of evolution in the 3’ UTRs of teleosts in general and cichlids in particular. The data suggest that 3’ UTRs might serve as important meta-regulators, regulators of other mechanisms governing post-transcriptional regulation, especially in groups like cichlids that have undergone extremely fast rates of phenotypic diversification and speciation.

**Electronic supplementary material:**

The online version of this article (10.1186/s12864-018-4821-8) contains supplementary material, which is available to authorized users.

## Background

Understanding the genomic mechanisms underlying adaptive radiations and phenotypic variation is one of the major objectives of evolutionary biology. Gene regulation has long been thought to be a particularly important component of phenotypic diversification [1]. For example, because of the inferred small amount of divergence in protein-coding genes, despite large phenotypic differences between humans and chimpanzees, it was suggested that mechanisms regulating differential gene expression must be playing a predominant role during our own adaptive evolution [2]. Due to major advances in the feasibility of generating fully-sequenced genomes [3], the importance of non-coding mechanisms during evolution can now be rigorously tested. As predicted, a large number of studies have found evidence for associations between gene regulatory mechanisms and phenotypic variation [4–7]. However, only a few studies [8] have examined the importance of three prime untranslated regions (3’ UTRs), that are located directly downstream of protein-coding DNA sequences, for adaptive divergence. These regions contain functional sequence elements that control mRNA stability [9], expression levels [10], and mRNA localization [11]. Therefore, they are undoubtedly important for gene regulation. In groups such as cichlid fishes, that are well known for their astonishing species richness, the evolutionary divergence of 3’ UTRs might provide an exceptionally important mechanism for their rapid evolution and diversity of adaptive phenotypes [12].

The 3’ UTR, the last component of messenger RNA (mRNA) to be transcribed in eukaryotes, starts directly after the stop codon and ends with a poly(A) tail. The 3’ UTR usually contains binding sites for RNA-binding proteins (RBPs) and small non-coding RNAs, which have important functional effects on the fate of mRNA. For instance, poly(A)-binding proteins (PABPs) stabilize the mRNA and facilitate translation by binding to the poly(A) tails [13]. 3’ UTRs are also the main target regions of microRNAs (miRNAs), ubiquitous non-coding small RNAs with a crucial role in fine-tuning gene regulation [14]. Mature miRNAs are incorporated into the RNA-induced silencing complex (RISC) and following interaction with target sites in the 3’ UTRs [15], which can cause translational repression and/or mRNA degradation [16]. We might expect that in groups like cichlid fishes that show exceptional phenotypic variation that there could be an increased abundance of miRNA targets in 3’ UTRs.

Since 3’ UTRs are a particularly important part of the genomic machinery, we might also expect that purifying selection in these regions could prevent the incorporation of non-essential elements. As a major source of genomic (especially non-coding region) variation [17, 18], repetitive elements (repeats) can cause gene disruption [19], double-strand breaks [20], and gene expression changes [21], which might commonly be purified from the 3’ UTR regions [22, 23]. A lower abundance of repeats in the 3’UTRs as compared to the genome as a whole would provide evidence that there has been purifying selection in these regions [24].

The lengthening of 3’ UTRs might be especially important because it could permit an increase in the number of miRNA targets, contribute to the emergence of new cell types, and even increase morphological complexity during evolution [25, 26]. It has been shown that humans have much longer 3’ UTRs compared to other mammals [27]. Cichlids might also have longer 3’ UTRs as compared to other teleosts. Additionally, one defining characteristic of adaptively radiating groups like cichlids is an increase in the pace of trait divergence relative to other lineages [28]. Therefore, the rate of evolutionary change in 3’ UTR lengths might provide the genomic substrate for many adaptive radiations. If there were a causal relationship between 3’ UTR length, function, and phenotypic change, then rapid modification of 3’ UTR length during evolution could facilitate rapid phenotypic evolution. Therefore, we might expect in a clade that is rapidly diversifying phenotypically such as cichlid fishes, that the 3’ UTRs are exceptionally long and/or diversifying rapidly.

Although most gene regions could function more or less the same in all teleosts, one might expect that genes that deviate the most from average to play an outsized role during adaptive radiations [29]. For instance, we might expect 3’ UTRs that are exceptionally long or exhibit enhanced rates of evolutionary divergence in a group such as cichlid fishes to have played a role in their adaptive radiation. Therefore, we aimed to identify such 3’ UTRs and thereby make inferences about the mechanistic role of 3’ UTRs during cichlid divergence [30]. Gene ontology (GO) terms [31] and KEGG pathways [32] are widely used to describe the functions and biological processes of interesting genes. If particular groups of genes that appear to be outliers are known to function together in biological processes such as morphogenesis or gene regulation, this could provide an interesting insight into the role of 3’ UTRs during adaptive divergence.

Cichlid fishes are one of the most species-rich clades of teleost fishes and exhibit virtually unparalleled levels of phenotypic diversity [33, 34]. For instance, a huge component of that diversity, more than 2000 species, have originated in three East African Great Lakes within an extremely short period of time [35, 36]. Remarkable phenotypic diversity is also found in many cichlid traits including body color [37], body shape [38], jaws [39], lips [40], and visual systems [41], that all are adaptations to a multitude of ecological niches. The extremely fast speciation rates and diverse phenotypic novelties in cichlids could be explained by changes in both protein-coding genes and the gene regulatory system [42]. To date, rapid sequence evolution has been found in protein-coding regions linked to morphogenesis, vision, and pigmentation [42]. However, molecular changes in gene regulatory systems have not been as well studied although cichlids are rapidly becoming a model system of genome evolution and several cichlid genomes have been fully sequenced [42]. Therefore, examining how genomic divergence of regulatory regions in this group compares to that in other teleosts should provide novel insights into not only vertebrate genome evolution in general but also genome evolution in a classic model of adaptive radiation.

We used comparative genomics to test several hypotheses about the evolutionary patterns of 3’ UTR divergence in cichlid fishes as compared to other teleosts. We first quantified the length of all 3’ UTRs in five cichlid species as well as four other model teleosts and determined if 3’ UTR length of more than two thousand 1:1 orthologous genes was on average longer in cichlids. To examine the evidence for purifying selection in 3’ UTRs, we compared the abundance of repetitive elements in the genome as a whole as compared to that in all 3’ UTRs of all nine teleost genomes we analyzed. Then, we examined how cichlid 3’ UTRs compared to those of other teleosts in the average composition of repetitive elements and number of miRNA binding sites. For 1:1 orthologous genes, we also compared the evolutionary rate of change in 3’ UTR length for cichlids versus non-cichlids. We then examined in detail the top 5% of genes that had the longest 3’ UTRs and the top 5% that showed the most rapid evolutionary divergence of 3’ UTR length in cichlids to determine what genes and pathways in these fishes showed exceptional divergence in their 3’ UTRs.

## Results

### Statistics and distribution of 3’ UTRs in teleost fishes

**Fig. 1:**
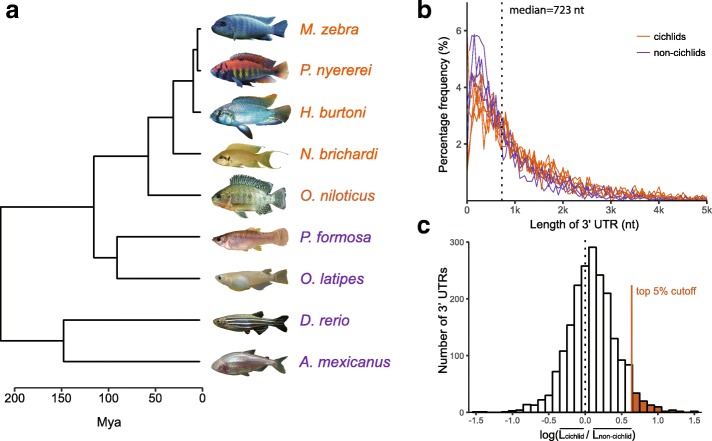
Length of 3’ UTRs in nine teleost fishes. **a** Phylogenetic tree of the focal nine teleost fishes with time-scale according to estimated divergence time from TimeTree [66]. The five species in red are cichlid species and the four species in purple are other model teleosts. **b** Frequency distribution of 3’ UTR length of nine teleost fishes for 2041 1:1 orthologous genes. In cichlids, there are fewer 3’UTRs that are shorter than the median length and more 3’UTRs that are longer than the median length. **c** Comparison of 3’ UTR length between cichlids and non-cichlids. The ratios of the mean length of 3’ UTR in cichlids to that in non-cichlids are calculated for 1:1 orthologous genes. These ratios were log_10_-transformed. In general, cichlids have longer 3’ UTRs than non-cichlids (one-sample t-test that the mean log_10_ ratio = 0.0; *p* <  0.001). The top 5% relatively longest 3’ UTRs in cichlids taken from these 2041 orthologous genes were further analyzed for enrichment of gene functions (GO terms and KEGG pathways). The images of *A. mexicanus*, *O. latipes*, *O. niloticus*, *M. zebra*, *N. brichardi* and *P. nyererei* were taken from Wikimedia Commons (http://commons.wikimedia.org/wiki/), while the images of *H. burtoni*, *D. rerio* and *P. formosa* were taken from FishBase (http://www.fishbase.org)

We examined gene structures of nine teleost fishes (Fig. 1a) based on species-specific transcripts from a large number of RNA-Seq experiments (Additional file 1: Table S1) and incorporated the structures into existing annotations using the PASA pipeline. In our updated PASA annotations, 212,135 genes and 563,263 transcripts with stop codons were annotated (Additional file 2: Table S2). The longest transcript of each gene was selected as the canonical transcript and the coding sequences and UTR sequences of canonical transcripts were extracted from the genomes according to the annotations (see Methods for details). Based on similarity searches, 184,810 genes were clustered into gene families and 2041 were identified as 1:1 orthologous genes that were present in all nine teleost genomes.

The 3’ UTRs occupied from 1.61 to 4.31 percentage of the teleost genomes (Table 1). *Haplochromis burtoni* had the largest combined total 3’ UTR length with 35,849,263 nucleotides (nt) as well as the longest mean length of 1552.66 nt, while *Astyanax mexicanus* had the smallest total 3’ UTR length of 19,162,274 nt and the shortest mean length of 836.05 nt. The frequency distribution of 3’ UTR length for all species is presented in the Additional file 3: Figure S1. The median 3’ UTR length of the nine teleost fishes was 795 nt. Cichlids tended to have longer 3’ UTRs in general. We also comparatively examined only the 3’ UTRs of 2041 1:1 orthologous genes and presented their distribution of lengths in Fig. 1b. We calculated the ratio of 3’ UTR length of 1:1 orthologous genes between cichlids and non-cichlids, and this ratio was on average significantly higher than the null expectation of 1.0 (the ratio’s value is 0 after log_10_-transformation) again indicating that 3’ UTRs were generally longer in cichlids as compared to the non-cichlids examined (Fig. 1c, *p* <  0.001).

**Table 1:** Summary statistics of 3’ UTR length of canonical transcripts in the nine teleost genomes

Species	Cichlids					Non-cichlids			
	*M. zebra*	*P. nyererei*	*H. burtoni*	*N.brichardi*	*O. niloticus*	*O. latipes*	*P. formosa*	*A. mexicanus*	*D. rerio*
Genome size (Mb)	860	830	831	848	928	870	749	1191	1372
Number of transcripts	23,910	22,358	23,089	21,921	25,191	21,979	24,901	22,920	25,866
Total 3’UTR length (Mb)	34.18	27.73	35.85	23.99	35.80	21.40	30.79	19.16	29.96
Percentage of 3’ UTR in genome	3.97	3.34	4.31	2.83	3.86	2.46	4.11	1.61	2.18
Mean length	1429	1240	1553	1094	1421	974	1237	836	1158
Median length	993	842	1087	690	970	669	812	516	691
25th percentile	410	317.25	463	194	381	277	333	175	270
75th percentile	1980	1727	2143	1552	1990.5	1334	1680	1133	1540
Maximum length	15,626	12,530	21,087	16,134	17,796	15,264	16,660	12,260	22,013

### Repetitive elements identification in 3’ UTRs

**Fig. 2:**
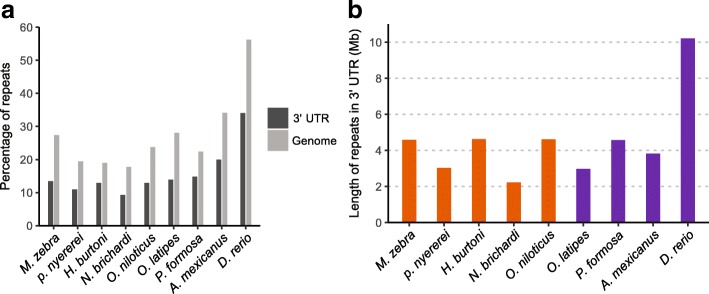
Repetitive elements in 3’ UTR. **a** The percentage of repetitive elements in 3’ UTRs and in the whole genome. In all nine teleost species, 3’ UTR regions contain a lower proportion of repetitive elements as compared to the genome as a whole (Wilcoxon signed-rank test, *p* = 0.004). **b** The total length of repetitive elements in 3’ UTRs

We identified repetitive elements (repeats) in 3’ UTRs and the whole genome respectively, to examine whether 3’ UTRs showed a lower percentage of repeats than the genome as a whole. Most of the repeats identified were transposable elements (TEs), including SINEs, LINEs, LTR retrotransposons and DNA transposons (Additional file 4: Table S3). All species had a lower proportion of repeats in the 3’ UTRs compared to their genomes as a whole. On average, the percentage of 3’ UTR that is made up by repeats for these teleosts ranged between 9 and 20% (against a percentage of repeats in the whole genome ranging between 18 and 38%). *Danio rerio* was exceptional as repeats made up 34% of its 3’ UTRs and *D. rerio* also had the highest proportion (52%) of the genome composed of repeats (Fig. 2a). However, for every single species, the proportion of the 3’ UTRs that was composed of repeats was significantly lower than the proportion of repetitive elements in their genomes as a whole (Wilcoxon signed-rank test, *p* = 0.004). To extend the comparison on the proportion of repeats among different genomic features, we additionally identified the repeats in the 5’ UTRs and coding regions in all of the species. The coding regions contained the lowest proportion of repeats (2–4%), while the 5’ UTRs contained a slightly lower proportion of repeats (from 6 to 11% depending on the target species) than the 3’ UTRs (Additional file 3: Figure S3; Additional file 4: Table S3). Despite the on average longer 3’ UTRs in cichlids, we did not find a clear pattern of increased repeats in cichlid 3’ UTRs (Fig. 2b). Importantly, the length of 3’ UTRs on average remained longer in cichlids after removing all repetitive elements (Additional file 3: Figure S2).

### miRNA targets prediction in 3’ UTRs

**Fig. 3:**
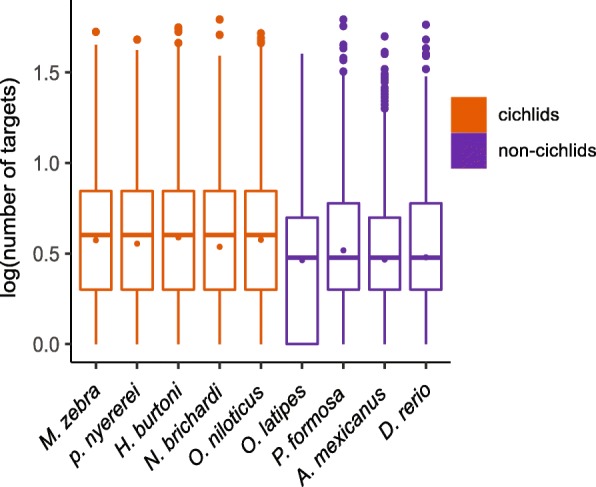
Predicted miRNA target sites per mRNA in 3’ UTRs. The box plots show the log_10_-transformed distribution of miRNA targets for the nine teleost fish species. The dots inside the boxes show the mean values. The outliers are shown by the dots outside the boxes. The maximum number of miRNA targets in a single 3’ UTR is 62, which can be found in one 3’ UTR in *N. brichardi* and one 3’ UTR in *P. formosa*. On average, cichlid fishes consistently have more miRNA target sites than non-cichlid fishes

Since miRNA targets are thought to be one of the most important functional motifs in 3’ UTRs [43], we searched for predicted miRNA targets in these nine teleosts 3’ UTR sequences. As species-specific miRNA data is not available and conserved miRNAs are expected to be unbiased in their presence, we used miRBase to screen all 3’ UTRs for the targets of 271 mature miRNAs that are conserved across vertebrates (Additional file 5: Table S4). From 42,611 to 83,210 targets per species were predicted in the 10,502 to 15,295 3’ UTRs per species (Additional file 6: Table S5). On average, there were more miRNA targets detected as well as more 3’ UTRs that were predicted to contain miRNA targets in cichlids. In the cichlid species examined, there were 4.9 to 5.6 miRNA targets per 3’ UTR versus a range of only 4.0 to 4.6 miRNA targets in the non-cichlid fishes (Fig. 3; Additional file 6: Table S5). Therefore, when compared to the other teleosts examined there was approximately one more miRNA target per 3’ UTR in cichlids.

### Evolutionary rate divergence in 3’ UTR length

**Fig. 4:**
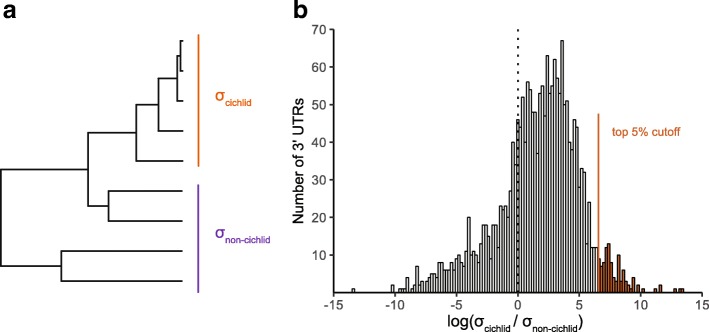
Evolutionary rate of 3’ UTR length. **a** The phylogeny used in rate estimation. The rates were estimated in cichlid and non-cichlid groups separately. **b** Comparison of evolutionary rate of 3’ UTRs between cichlids and non-cichlids. The ratios of the rate in cichlids to that in non-cichlids are calculated for 2.041 1:1 orthologous genes. These ratios were log_10_-transformed. In general, 3’ UTRs in cichlids have significant higher evolutionary rates than in non-cichlids (one-sample t-test that the mean log_10_ ratio = 0.0; p <  0.001). The top 5% relatively fastest evolving 3’ UTRs were further analyzed for enrichment of gene functions (GO terms and KEGG pathways)

Because rapid evolutionary change in 3’ UTR length might be associated with substantial phenotypic divergence, we estimated the evolutionary divergence rate of 3’ UTR length under a time-calibrated phylogenetic framework (Fig. 4a). For each of the 2041 1:1 orthologous genes identified, rates were estimated both in the group of cichlids and the group of non-cichlids separately. Then, the ratio between these rates was calculated by dividing the cichlid rate by the non-cichlid rate. In general, cichlids had a significantly higher evolutionary rate of 3’ UTR divergence than non-cichlids (Fig. 4b, one sample t-test, *p* <  0.001).

### Gene set enrichment analysis of long and rapid-evolving 3’ UTRs

Next, we selected two sets of genes to further examine the hypothesis that 3’ UTRs might be important for particular aspects of cichlid diversification by conducting GO-enrichment analyses for (1) the genes with relatively longest 3’ UTRs in cichlids and (2) genes that exhibited relatively rapid-evolving 3’ UTRs in cichlids. A top 5% cutoff was applied to 1:1 orthologous genes and thereby 101 genes were selected for each set. Both of these relatively longest and fastest evolving 3’ UTRs were then subjected to enrichment analysis (GO terms and KEGG pathways). There were 47 genes shared between the “length” set and the “rate” set, which meant 46.5% of them overlapped. By comparing proteins of *D. rerio* in the Ensembl database (release 91), only one of these genes did not have a match (Additional file 3: Figure S4; Additional file 7: Table S6). The enrichment analysis revealed that 13 GO terms were significantly enriched (Table 2). A number of GO terms were related to both ribosome and translation, a pattern that was confirmed by the enriched KEGG “Ribosome” pathway (*p* <  0.001).

**Table 2:** Gene set enrichment analysis of relatively longest and fastest evolving 3’ UTRs in cichlids. Significant enriched GO terms are presented. CC: cellular component; BP: biological process; MF: molecular function; KEGG: KEGG pathway

GO category	GO term	Significant *p*-value		
		“length”	“rate”	“union”
CC	macromolecular complex	–	< 0.001	–
CC	mitochondrial matrix	0.046	–	–
CC	intracellular ribonucleoprotein complex	–	< 0.001	–
CC	Ribosome	< 0.001	< 0.001	0.001
CC	ribosomal subunit	< 0.001	–	–
BP	cellular biosynthetic process	–	–	0.046
BP	nucleic acid phosphodiester bond hydrolysis	0.037	–	–
BP	peptide metabolic process	< 0.001	< 0.001	< 0.001
BP	primary metabolic process	–	0.019	–
BP	Translation	< 0.001	< 0.001	–
MF	nuclease activity	0.013	–	–
MF	RNA binding	–	< 0.001	–
MF	structural constituent of ribosome	< 0.001	< 0.001	< 0.001
KEGG	Ribosome	< 0.001	< 0.001	< 0.001

## Discussion

The divergence of 3’ UTRs could play important roles in both genomic as well as phenotypic evolution. Therefore, we characterized 3’ UTRs in the genomes of nine teleost fishes and documented patterns of genome-wide 3’ UTR divergence in cichlid fishes compared to non-cichlid fishes. We found that 3’ UTRs in cichlid fish genomes are on average longer than those in other fish lineages. The relative paucity of repetitive elements in 3’ UTRs as compared to the whole genome in all of the teleosts examined speaks to the functional importance of this region and suggests purifying selection could be operating to keep transposable elements and other insertions out of 3’ UTRs. Moreover, the on average longer 3’ UTRs in cichlids is associated with a greater number of miRNA targets. There also appears to be a higher average evolutionary rate of divergence in 3’ UTR sequence length in cichlids as compared to other teleosts. Additionally, analysis of gene function on both the longest and fastest-evolving cichlid 3’ UTRs showed a strong functional bias towards ribosome-related pathways and translation. These associations suggest that macro-evolutionary divergence in 3’ UTRs in cichlids might be influencing the core post-transcriptional regulation machinery in these rapidly diversifying fishes. In general, our results support a role of 3’ UTRs as meta-regulators, regulators of other gene regulatory mechanisms, in groups undergoing exceptional speciation and adaptive phenotypic diversification.

The lengths of 3’ UTRs likely represent compromises among a number of factors. While 3’ UTRs are not translated into amino acids, the process of transcription is energetically consuming. We, therefore, expected that most 3’ UTRs should be relatively short and this was supported in our analyses (Additional file 3: Figure S1). The length of a 3’ UTR is also known to be important because it is associated with gene expression levels [44]. Longer 3’ UTRs usually contain more functional motifs and are associated with lower gene expression levels [45]. For the nine teleost fishes examined, their overall 3’ UTRs had a median length of 795 nt, while the 3’ UTRs of only 1:1 orthologous genes had a median length of 723 nt. It appears that cichlids have more 3’ UTRs longer than the median compared to non-cichlids (Fig. 1b; Additional file 3: Figure S1) and it is confirmed by comparing 1:1 orthologous genes that 3’ UTRs on average were longer in cichlids than in non-cichlids (Fig. 1c), which could indicate a greater potential for a diversity of functions in cichlid 3’ UTRs.

Repetitive elements (repeats) are mobile genetic units that can shape a number of aspects of eukaryotic genome architecture including 3’UTR length [46]. It has been proposed that repeats are one of the main contributors of non-coding variation among different species [47, 48] and their expansion history and impact on genome diversity both between and within lineages has also been documented in many fish [42, 49, 50]. The abundancy of repeats is uneven across the genome, and this variation could be associated with different levels of selective constraints acting on different components of the genome [51]. In each of the nine focal species, we identified the abundance of repeats in the coding regions, 5’ UTRs, 3’ UTRs and in the genome as a whole. Our results show that coding regions are made up of only 2–4% of repeats, and this is not surprising considering the strong selective pressures on protein coding genes. Additionally, in all species, both 5′ and 3’ UTRs contain smaller proportions of repeats than the genome as a whole, but higher than the coding regions, which is consistent with the hypothesis that purifying selection acts against the presence of repeats in UTRs [52, 53]. Focusing on 3’ UTRs, *Dario rerio* stood out from the other teleosts, in that it has the largest proportion of repeats in its genome as well as in its 3’ UTRs. This is likely due to the documented genome-wide expansion of repeats in this species [54, 55]. *Astyanax mexicanus* has the second largest proportion of repeats in the 3’ UTRs and this was also mirrored in the proportion of repetitive elements in its genome. Less variation was present in the other fish species investigated in this study, and variation of 3’ UTR length among different taxa did not appear to be the result of an expansion of repeats (Fig. 2b). This implies other mechanisms are likely driving the evolution of 3’ UTR length divergence among teleosts.

The on average lengthening of 3’ UTRs in cichlids could be associated with a larger number of functional motifs, such as RNA binding sites and miRNA targets. Indeed, we identified about one more miRNA target per 3’ UTRs in cichlids compared to the other fish lineages (Fig. 3; Additional file 6: Table S5). Also, we found that more mRNAs are targeted by miRNAs and more mRNAs have multiple targets in cichlids (Additional file 6: Table S5). This could have consequences for cichlid diversification as more potential miRNA-mRNA functional pairs in cichlids could enable these fishes to regulate gene expression spatially and temporally to an exceptional degree [8, 56].

Both coding and non-coding divergence likely contribute to diversification. Accelerated sequence evolution of particular coding regions in cichlids has been considered to be associated with their adaptive radiation [42]. Interestingly, we also detected that 3’ UTRs have on average evolved significantly faster in cichlids than in other teleosts (Fig. 4b). The rapid evolution of 3’ UTRs within cichlids implicates this part of the post-transcriptional regulatory system in structuring the exceptional phenotypic diversification of cichlids [57, 58].

Particular subsets of 3’ UTRs might be especially important during adaptive diversification. We, therefore, examined two sets of evolutionary outliers for further gene function enrichment analysis: (1) genes with relatively longest 3’ UTRs and (2) genes with the relatively fastest evolving 3’ UTRs. The influence of these subsets of genes on gene expression could play an important role in the diversification of cichlids [59, 60]. Surprisingly, the genes in these two datasets most often have functions related to ribosomal structures and related pathways. The ribosome is the main organelle in the process of translation, the final step of gene expression after post-transcriptional regulation. In addition, GO analyses suggested “RNA binding” was also one of the main functions of these 3’ UTRs. The evolution of 3’ UTRs in cichlids appears to have most affected the core of cellular life, post-transcriptional regulation and protein synthesis, rather than particular genes such as morphogens or opsins that more directly influence individual phenotypes. Although ribosomal proteins are highly conserved in coding regions because of their crucial functions in protein synthesis, it has been shown that the diversity and differential expression of ribosomal components, known as specialized ribosomes, can selectively translate certain sets of genes [61]. This new level of gene regulation can be further linked to many phenotypes, such as development [62] and disease [63, 64]. The fast evolution of 3’ UTRs of ribosomal genes in cichlids could provide the potential for specialized expression of specific ribosomal genes both spatially and temporally, which could be an important post-transcriptional regulatory mechanism that has contributed to the adaptation and diversification of cichlid fishes.

## Conclusions

Our results reveal novel patterns of evolution and potential functional differences in the 3’ UTRs of teleosts in general and of cichlid fishes in particular. It has long been argued that because of the relatively small amount of protein-coding divergence, but large phenotypic differences found between groups like humans and chimpanzees, that differential gene expression and associated regulatory mechanisms must play an important role in adaptive evolution [2]. Our analyses suggest that what might be most critical during adaptive divergence is not only the direct regulators of gene expression but the regulators of these more direct regulators. By preferentially influencing divergence in other regulatory mechanisms such as the ribosome and other aspects of the post-translational machinery during the adaptive radiation of groups like cichlid fishes, divergence in 3’ UTRs could serve as important genomic meta-regulators.

## Methods

### Data collection

The NCBI database [65] was searched for genomic and transcriptomic data of teleost fishes. In order to obtain high-quality annotations of 3’ UTRs, we applied the following minimal criteria for including species into our data set: a) de novo genome is assembled; b) species-specific gene models are predicted; c) transcriptomic data from numerous tissues is available. These stringent criteria enabled us to carry out comprehensive gene and isoform discovery in each species, thus minimizing the potential bias due to uneven gene and isoform representation across species. Nine teleost fishes matched these criteria and were included in our analyses. Specifically, genome assemblies, annotations and RNA sequencing (RNA-Seq) data for five cichlid fishes (*Neolamprologus brichardi*, *Pundamilia nyererei*, *Maylandia zebra*, *Oreochromis niloticus*, *Haplochromis burtoni*) and four non-cichlid teleost fishes (*Astyanax mexicanus*, *Danio rerio*, *Oryzias latipes*, *Poecilia formosa*) were examined (Additional file 1: Table S1). It is important to note that the nine selected species underwent the same number of whole genome duplication (WGD) events and are not descended from a lineage containing an extra round of WGD after the teleost-specific WGD. This gave us more confidence in detecting the correct orthology among genes (see below). To place these species in a comparative framework, a time-calibrated phylogenetic tree of these nine teleost fishes was acquired from TimeTree [66].

### Annotation of 3’ UTRs with the PASA pipeline

The number of genomes of teleost fishes that are available is increasing quickly, but the comparison of 3’ UTRs across genomes remains challenging. Although the annotations of gene models are usually published with the genomes, the quality of these annotations is often uneven because different pipelines have been applied in each species. Moreover, the UTRs are often poorly described since most annotations typically focus on the protein-coding regions. Thus, the PASA v2.0.2 annotation pipeline [67] was employed to incorporate gene structures, including UTRs, into existing gene annotation, based on RNA-Seq data.

A comprehensive workflow was used to integrate the available transcriptomes into existing annotations. For each species, RNA-Seq data from eight to eleven tissues and different developmental stages (Additional file 1: Table S1) was used. First, transcripts for each species were generated by combining de novo assembly and genome-guided assembly. The RNA-Seq reads were de novo assembled using Trinity v2.4.0 [68] using default parameters. Moreover, the RNA-Seq raw reads were mapped to the corresponding genome using TopHat v2.1.0 [69] also with default parameters and then assembled into transcripts using the genome-guide model implemented in Trinity. Then, the gene structures were identified according to TopHat mapping results using Cufflinks v2.2.1 [70]. Lastly, the original annotation, transcripts, and Cufflinks gene structures were imported into the PASA annotation pipeline. The annotation output from the first PASA run, transcripts, and Cufflinks gene structures were then used in an additional run of PASA to further refine the gene structure and get the final annotations for each of the species (the updated gene models have been uploaded to the Dryad Digital Repository (doi:10.5061/dryad.4581cm37).

### Retrieval of coding and UTR sequences

From the PASA annotations, we selected only transcripts that contained stop codons to ensure that the transcripts were not degraded during RNA sequencing. For genes with alternative splicing variants, we selected only the transcript with the longest coding region as the canonical transcript. The sequences of coding regions and UTRs of the canonical transcripts were then extracted from the corresponding genome according to the PASA annotation. The frequency distributions of 3’ UTR lengths, calculated using 50 nt windows, were built for each of the nine teleost fishes.

### Gene clustering

Protein sequences of canonical transcripts were extracted from the PASA annotations and genomes. All-against-all BLASTP v2.2.31+ [71] was employed to find the potential homologous genes with an E-value cutoff of 1e^− 10^. The high-scoring segment pairs (HSPs) from BLASTP output were conjoined by using Solar v0.9.6 [72]. The similarity of genes was measured by the bit-score. Most similar genes were clustered to gene families using hcluster_sg v0.5.1, a hierarchical clustering algorithm from the Treefam pipeline [73], with the parameters “-w 5 -s 0.33 -m 100000”.

### Identification of repetitive sequences (repeats)

The species-specific libraries of repeat families were identified by applying RepeatModeler v1.0.8 [74] on the genome of each species. RepeatMasker v4.0.6 [75] was used to mask interspersed repeats and low complexity DNA sequences in the genomes. To compare the proportion of repetitive elements in different genomic regions across the focal species, sequence repeats were annotated in the 3’ UTRs, 5’ UTRs, and in the coding regions.

### Prediction of miRNA target sites.

Sequences of mature miRNAs from teleost fishes were retrieved from miRBase v21 [76]. Since miRNAs are usually conserved across taxa [77] and species-specific miRNAs are not present in the database for all species in this study, we extracted 271 unique mature miRNAs conserved among vertebrates (Additional file 5: Table S4) for the target prediction. The miRNA target sites of 3’ UTRs were predicted using miRanda v3.3a [78] with parameters “-en -20 -strict”, which set the minimal free energy as − 20 kcal/mol and required 5′-seed [79] in the mRNA-miRNA matches.

### Evolutionary rate of 3’ UTRs

The evolutionary rates of 3’ UTR length for all 1:1 orthologous genes were examined using the R package “OUwie v1.50” [80] under the Brownian motion model. Rates were estimated on the time-calibrated phylogenetic tree for two groups. Specifically, the rates within the cichlid clade and in the non-cichlid outgroup were estimated separately. Then, the ratio between the estimated rates in cichlids and in non-cichlids was calculated and a log transformation was applied to the ratio to normalize the data for further analyses.

### Gene set enrichment analysis

Enrichment analysis was performed on two data sets taken from the analyses on the 1:1 orthologous genes. We examined the upper 5% percentile distribution of the genes that had relatively longest 3’ UTRs and the upper 5% percentile distribution form the 3’ UTRs showing the relative fastest evolutionary rate in cichlids. To identify significantly over-represented gene ontology (GO) terms and KEGG pathways in the identified genes (test sets) when compared to the whole gene set (baseline set), we used a Fisher’s exact test implemented in g:Profiler [81]. To avoid any bias due to the different quality of the genome annotations, we carried out different analyses using as baseline the *O. niloticus* and the *D. rerio* gene sets, the best-annotated cichlids and non-cichlids genomes, respectively. The sequence distribution of the GO terms for the genes in the test sets was observed graphically using a multilevel pie representation method as implemented in Blast2GO v4.1.5 [82].

### Statistics

We performed three types of comparisons and performed different statistical analyses based on the type of comparisons made with the teleost 3’ UTRs. When we compared different values within a species, such as the proportion of repetitive elements in the whole genome versus the 3’ UTRs, we treated the species as the unit of replication. For the comparisons of length and evolutionary rates involving large numbers of individual genes, we treated these genes as independent data points for comparisons between cichlids and non-cichlids. When we compared the average values, such as miRNA targets, in cichlids versus non-cichlids, we did not perform any statistical analyses as these groups and their aggregate trait values were not phylogenetically independent of one another, and therefore, in these cases, we only reported the patterns.

## Additional files

Additional file 1: **Table S1.** Genomic and transcriptomic data of the nine teleost fish species included in this study. (XLSX 11 kb)

Additional file 2: **Table S2.** Comparison of annotations for the nine teleost fish species. (XLSX 10 kb)

Additional file 3: **Figure S1.** Frequency distribution of 3’ UTR length in nine teleost species. **Figure S2.** Mean length of 3’ UTRs without repeats. **Figure S3.** Different classes of repetitive elements in different genomic components in nine teleost fish species. **Figure S4.** Multi-level pie charts of GO terms of selected genes. (PDF 1211 kb)

Additional file 4: **Table S3.** Percentage of repetitive elements in 3’ UTRs, 5’ UTRs, coding regions and in the whole genome. (XLSX 13 kb)

Additional file 5: **Table S4.** Name and sequence of the 271 teleost miRNAs conserved in vertebrates. (XLSX 18 kb)

Additional file 6: **Table S5.** Predicted miRNA targets in the focal nine teleost fish species. (XLSX 11 kb)

Additional file 7: **Table S6.** GO annotations of the selected genes with relatively longest and fastest-evolving 3’ UTRs in cichlid fishes. (XLSX 35 kb)

## Abbreviations

3’ UTRs: Three prime untranslated regions
GO: Gene ontology
HSPs: High-scoreing segment pairs
miRNAs: MicroRNAs
mRNA: Messenger RNA
Nt: Nucleotide
PABPs: Poly(A)-binding proteins
RBPs: RNA-binding proteins
Repeats: Repetitive elements
RISC: RNA-induced silencing complex
RNA-Seq: RNA sequencing
TEs: Transposable elements
WGD: Whole genome duplication
