# Supplementary material for: Evolutionary divergence of 3’ UTRs in cichlid fishes
Source: BMC Genomics. 2018 Jun 5;19:433. doi: 10.1186/s12864-018-4821-8 (PMC5987618; doi:10.1186/s12864-018-4821-8)
Supplement: Supplementary file 3 — Figure S1. Frequency distribution of 3’ UTR length in nine teleost species. Figure S2. Mean length of 3’ UTRs without repeats. Figure S3. Different classes of repetitive elements in different genomic components in nine teleost fish species. Figure S4. Multi-level pie charts of GO terms of selected genes. (PDF 1211 kb) [file 12864_2018_4821_MOESM3_ESM.pdf]

Evolutionary Divergence of 3' UTRs in Cichlid Fishes

Peiwen Xiong[1], C. Darrin Hulsey[1], Axel Meyer[1, 2], Paolo Franchini[1, *]

[1]Chair in Zoology and Evolutionary Biology, Department of Biology, University of Konstanz, 78457 Konstanz, Germany
[2]Radcliffe Institute for Advanced Study, Harvard University, Cambridge 02138, MA, USA

[*]Correspondence: paolo.franchini@uni-konstanz.de

Additional file 1: Figure S1-S4

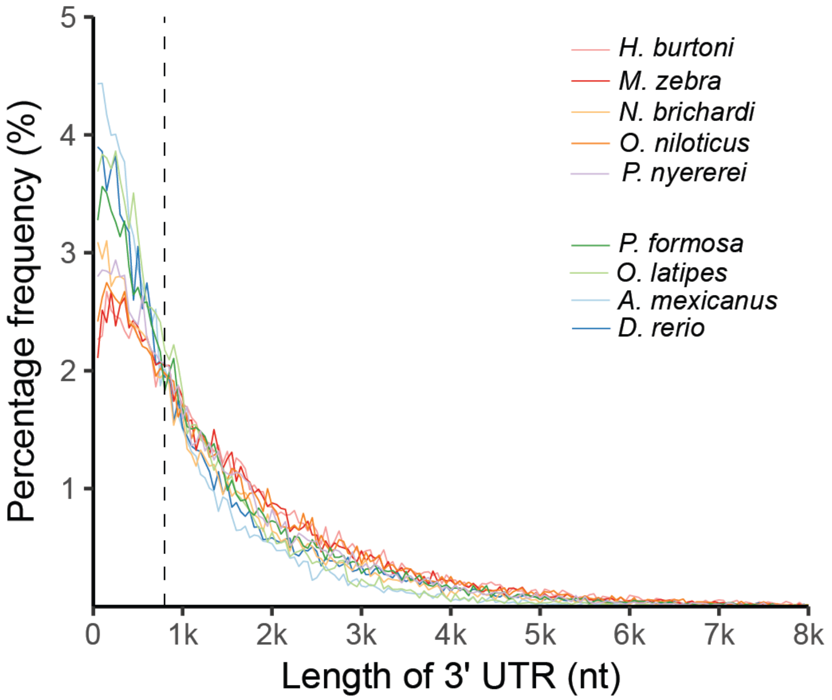

Figure S1. Frequency distribution of 3' UTR length of all genes in nine teleost species.

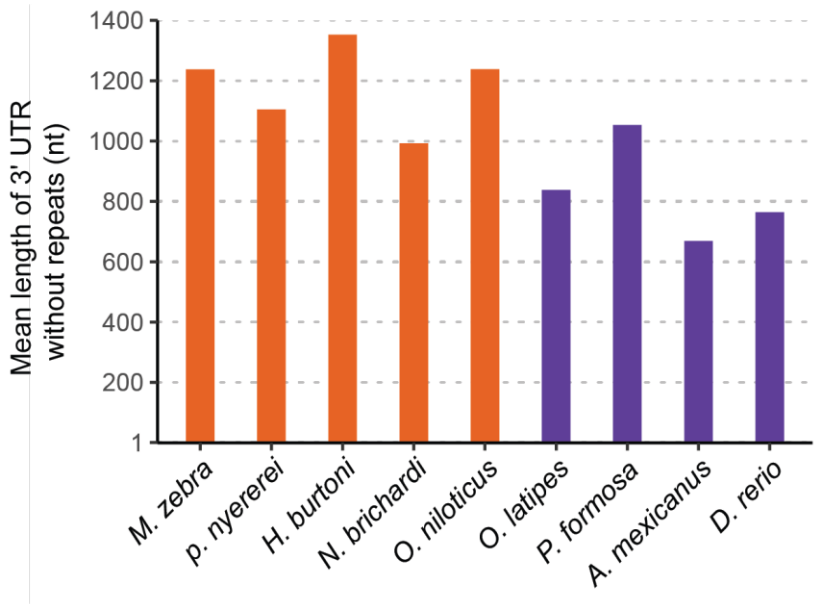

Figure S2. Mean length of 3’ UTRs after removing repetitive elements.

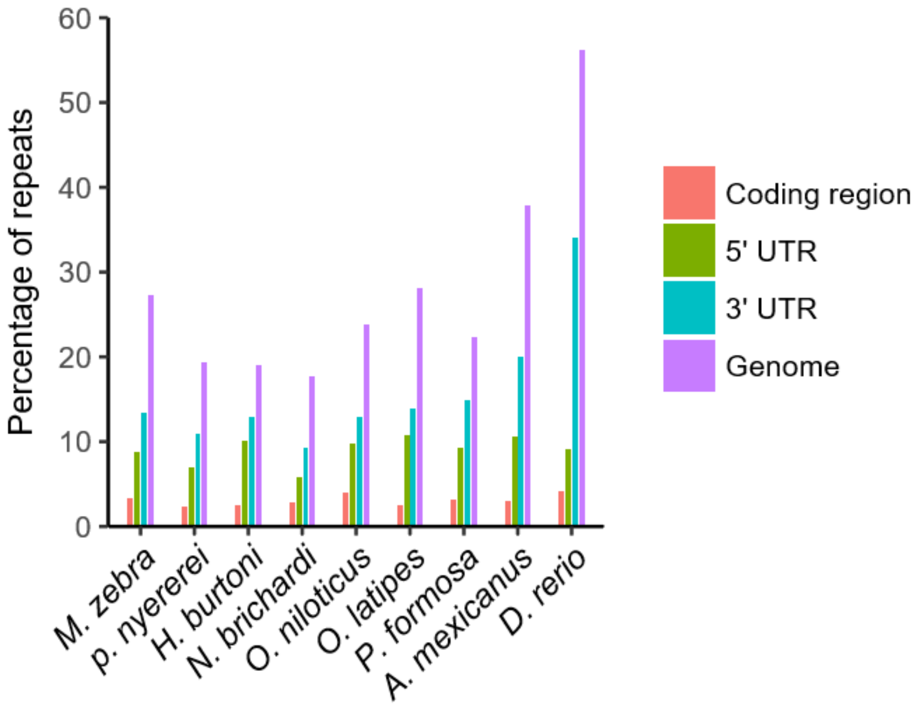

Figure S3. Different classes of repetitive elements in different genomic components in nine teleost fish species.

a

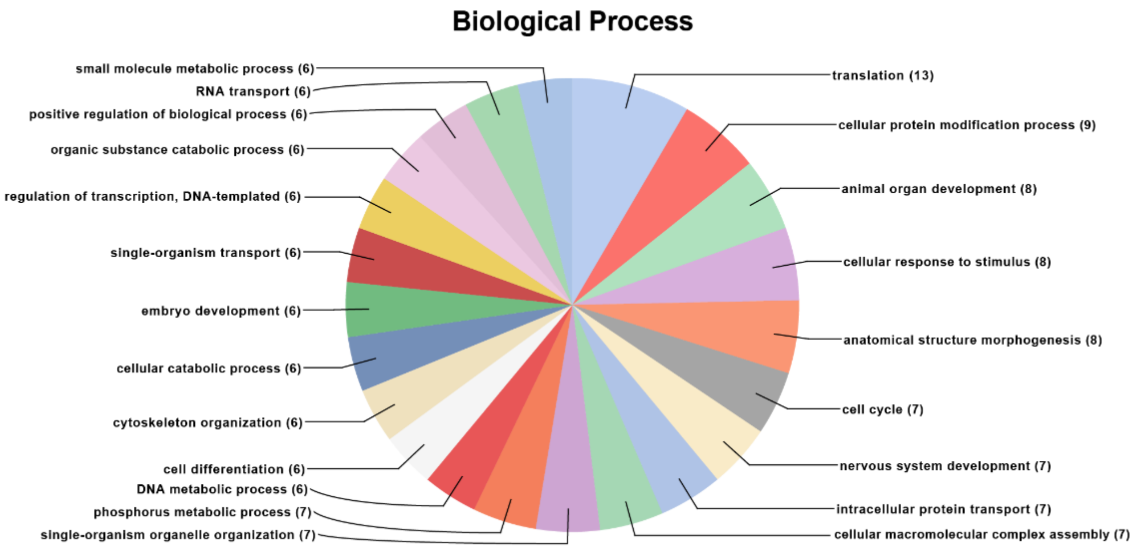
Biological Process
small molecule metabolic process (6)
RNA transport (6)
positive regulation of biological process (6)
organic substance catabolic process (6)
regulation of transcription, DNA-templated (6)
single-organism transport (6)
embryo development (6)
cellular catabolic process (6)
cytoskeleton organization (6)
cell differentiation (6)
DNA metabolic process (6)
phosphorus metabolic process (7)
single-organism organelle organization (7)
translation (13)
cellular protein modification process (9)
animal organ development (8)
cellular response to stimulus (8)
anatomical structure morphogenesis (8)
cell cycle (7)
nervous system development (7)
intracellular protein transport (7)
cellular macromolecular complex assembly (7)

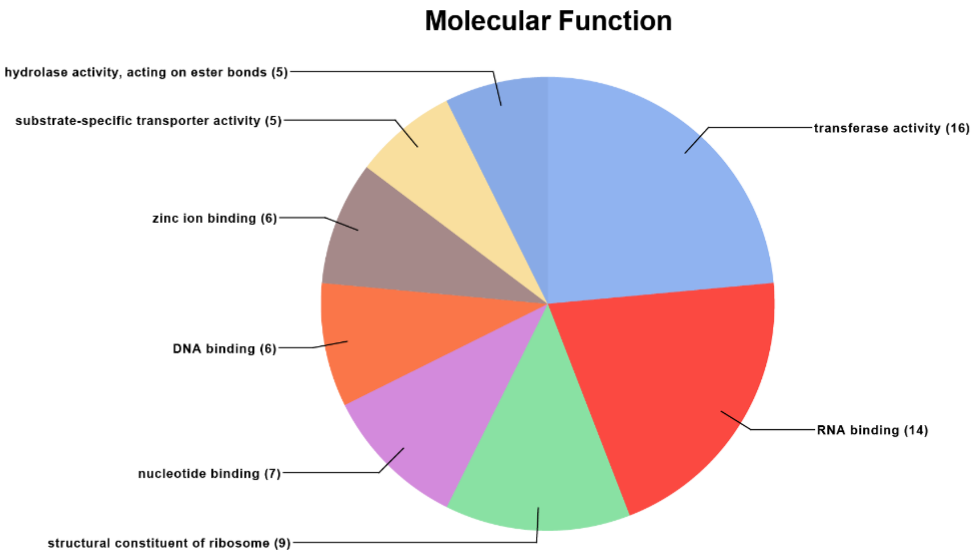
Molecular Function
hydrolase activity, acting on ester bonds (5)
substrate-specific transporter activity (5)
zinc ion binding (6)
DNA binding (6)
nucleotide binding (7)
structural constituent of ribosome (9)
transferase activity (16)
RNA binding (14)

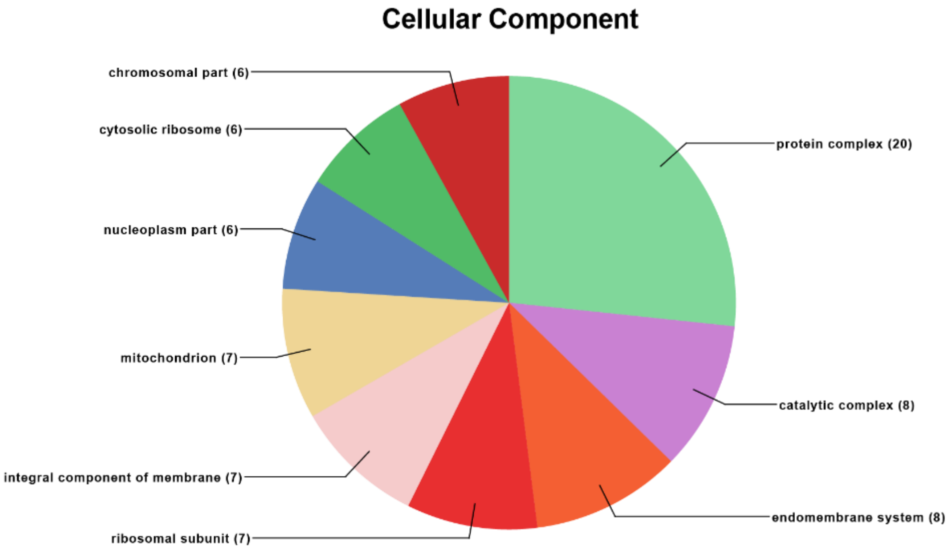
Cellular Component
chromosomal part (6)
cytosolic ribosome (6)
nucleoplasm part (6)
mitochondrion (7)
integral component of membrane (7)
ribosomal subunit (7)
protein complex (20)
catalytic complex (8)
endomembrane system (8)

b

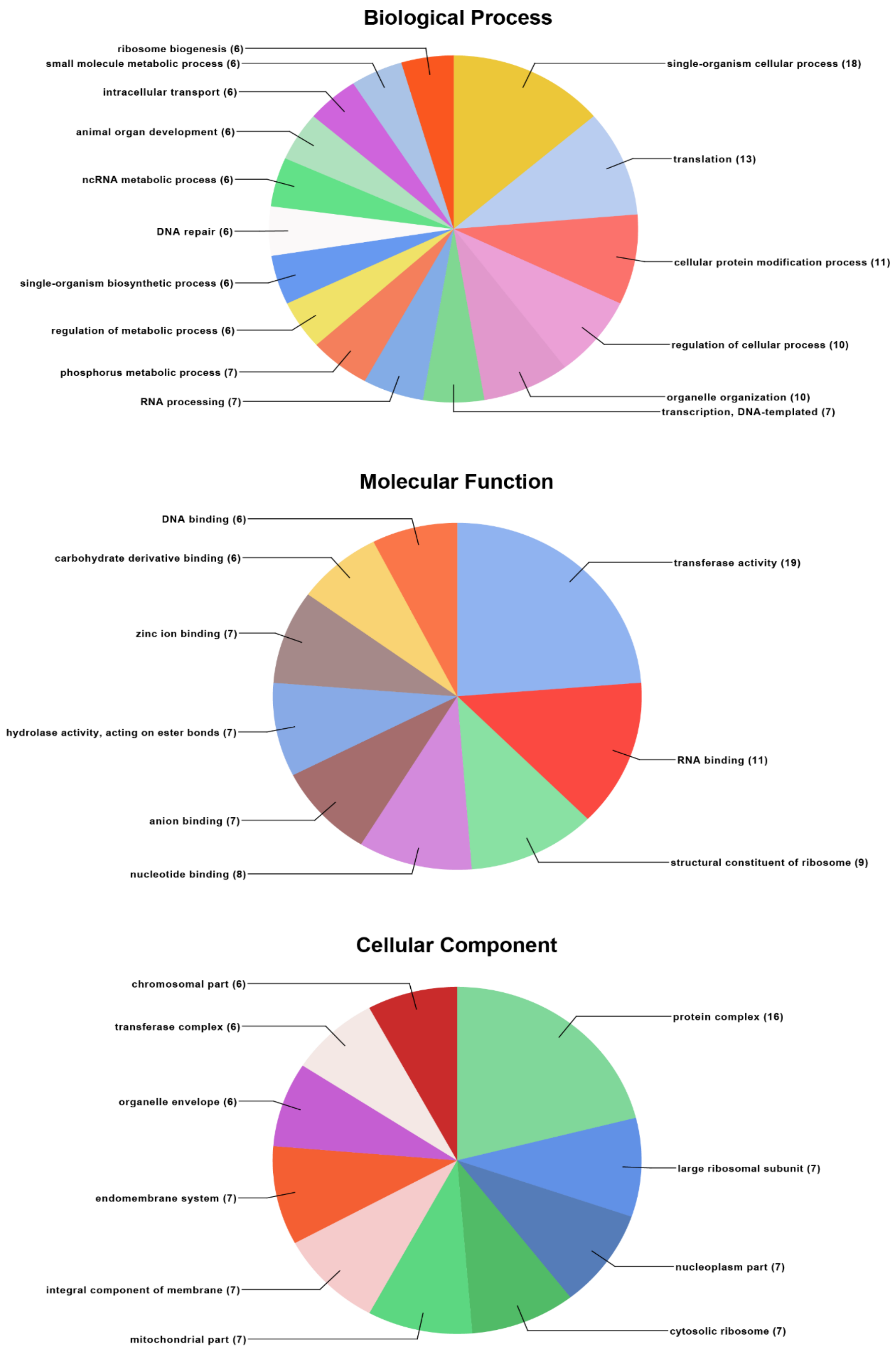

Figure S3. Multi-level pie charts of GO terms of selected genes. Relatively **a** fast-evolving and **b** long 3' UTRs in cichlids grouped in the three main functional categories: biological process, molecular function and cellular component. The multi-level pie charts were created using Blast2GO v4.1 (Cones *et al*. 2005).
